# Extending the fossil record of late Oligocene non-biting midges (Chironomidae, Diptera) of New Zealand

**DOI:** 10.7717/peerj.18893

**Published:** 2025-02-21

**Authors:** Viktor O. Baranov, Jörg U. Hammel, Daphne E. Lee, Alexander R. Schmidt, Uwe Kaulfuss

**Affiliations:** 1 Estación Biológica de Doñana, Consejo Superior de Investigaciones Científicas, Sevilla, Spain; 2 Institute of Materials Physics, Helmholtz-Zentrum Hereon, Geesthacht, Germany; 3 Department of Geology, University of Otago, Dunedin, New Zealand; 4 Department of Geobiology, University of Göttingen, Göttingen, Germany; 5 Department of Animal Evolution & Biodiversity, University of Göttingen, Göttingen, Germany

**Keywords:** Chironomidae, Fossil insects, Palaeoecology, Pomahaka Formation, Zealandia

## Abstract

**Background:**

The modern chironomid fauna of New Zealand is diverse, highly endemic and reflects a complex biogeographical history. This fauna has been important for developing phylogenetic and biogeographic concepts including Brundin’s writings on transantarctic relationships but until now the fossil record to support these reconstructions has been very limited. Here we describe the first fossil species of Chironomidae, subfamily Orthocladiinae, from New Zealand, based on inclusions in amber from the late Oligocene Pomahaka Formation of the South Island.

**Methods:**

We examined newly excavated fossil tree resin (amber) from the late Oligocene Pomahaka Formation in southern New Zealand for inclusions. Amber pieces containing chironomids were prepared and morphologically investigated using light-microscopy and µCT-scanning. Specimens were taxonomically evaluated using identification keys for modern adult chironomid midges. Habitus and key morphological features of each specimen were documented photographically and/or by line drawings.

**Results:**

Thirteen Chironomidae specimens from Pomahaka amber were identified as members of the subfamily Orthocladiinae Kieffer. *Bryophaenocladius zealandiae* sp. nov. Baranov is the first Southern Hemisphere fossil of the genus. *Bryophaenocladius* Thienemann, 1934 is absent from the extant fauna of the main islands of New Zealand; however, it may be present on the subantarctic Auckland Islands. Two incompletely preserved specimens are described as Morphotype 1 cf. *Bryophaenocladius zealandiae*. Based on a male adult, *Pterosis extinctus* sp. nov. Baranov is described as the first fossil record of the extant genus *Pterosis* Sublette and Wirth, today represented by a single endemic species on the New Zealand subantarctic Auckland Islands and Campbell Island. Two female adult specimens are described as Morphotype 2 cf. Metriocnemini. The new fossils of the genera *Bryophaenocladius* and *Pterosis* belong to chironomid taxa requiring terrestrial or semi-aquatic habitats for larval development, supporting the notion of a humid forest swamp paleoenvironment for the Pomahaka amber source forest.

## Introduction

Non-biting midges (Chironomidae) have historically served as a model group for the development of both modern phylogenetic analysis and historical biogeography (Hennig, 1960; Brundin, 1966). Studies of the extant Chironomidae fauna of New Zealand have played a major role in understanding transantarctic vicariance patterns (Brundin, 1966; Krosch & Cranston, 2013). In particular, phylogenetic studies of the Podonominae, southern Diamesinae and austral Orthocladiinae were seminal for understanding vicariance patterns caused by the break-up of Gondwana (Brundin, 1966; Krosch et al., 2011; Krosch & Cranston, 2013). The fossil record of New Zealand’s Chironomidae fauna is therefore very important for understanding biogeographic patterns in the Southern Hemisphere (Schmidt et al., 2018; Baranov, Haug & Kaulfuss, 2024).

Our knowledge of the fossil history of Chironomidae in New Zealand has been very limited, so far, despite significant studies mentioned above. Schmidt et al. (2018) reported four specimens of *Bryophaenocladius* Thienemann (Orthocladiinae) from Oligocene amber from the South Island, which are included in our descriptions herein. Baranov, Haug & Kaulfuss (2024) described three morphotypes of immature Chironomidae from Early Miocene lake sediments at Foulden Maar on the South Island. Subfossil records of Chironomidae include the larvae of *Corynocera duffi* Deevey, 1955 from Holocene swamp deposits in Canterbury, South Island (Deevey, 1955) and numerous other chironomid taxa identified from various Holocene sites on South Island (Schakau, 1991; Woodward & Shulmeister, 2007; Dieffenbacher-Krall et al., 2008).

Considering this limited fossil record, it is difficult to improve our understanding of the evolutionary history of Chironomidae in New Zealand, particularly for the subfamily Orthocladiinae. Thus, any additional deep time records of this subfamily from New Zealand are of great value. In this study, we describe two new species of Orthocladiinae from Oligocene amber from the South Island of New Zealand. These new discoveries add valuable knowledge to our understanding of the past diversity and historical biogeography of Orthocladiinae in New Zealand.

## Geological setting

**Figure 1:**
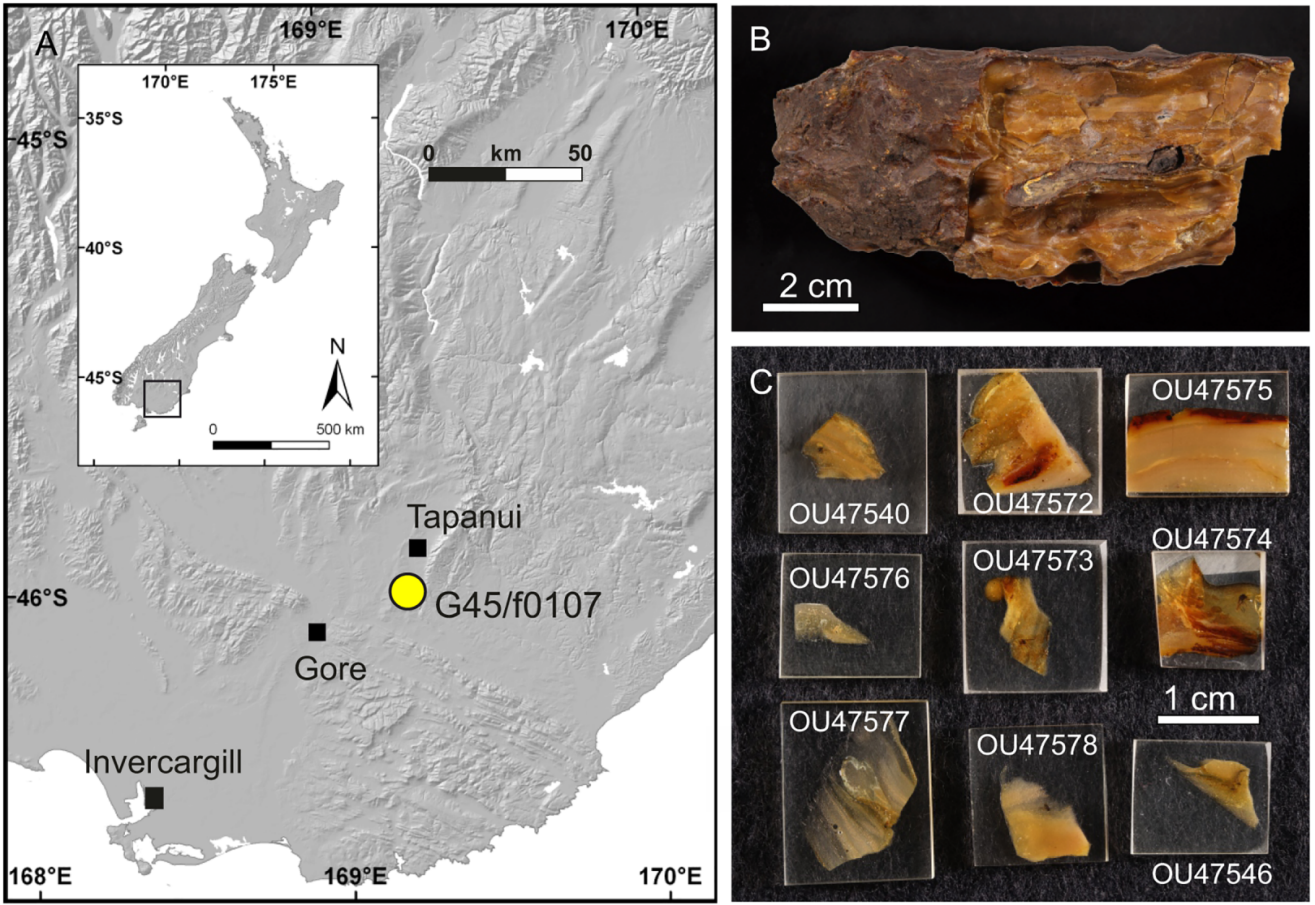
Late Oligocene Pomahaka amber. (A) Map of amber locality G45/f0107 near Tapanui, southern New Zealand. (B) Typical appearance of layered, fossiliferous Pomahaka amber. (C) Epoxy-embedded pieces of Pomahaka amber with newly discovered Chironomidae inclusions.

The Chironomidae specimens studied here are inclusions in amber from the estuarine late Oligocene Pomahaka Formation in southern New Zealand. Fossiliferous amber was collected from a lignite bed and associated carbonaceous mudstone in a temporary excavation pit on private farmland near Pomahaka River approx. 12 km south of Tapanui (46.04450°S, 169.22292°E) (Fig. 1). The locality is registered as G45/f0107 in the New Zealand Fossil Record File (GNS Science & Geological Society of New Zealand, 2024). Fourier-transform infrared spectroscopy analysis of amber from the site indicates an araucarian, *Agathis*-like parent plant, which is supported by finds of araucarian wood and abundant pollen of *Araucariacites australis* Cookson, 1947 in Pomahaka Formation sediments (Pocknall, 1982; Lee et al., 2009; Kaulfuss et al., 2024). Within the lignite and underlying carbonaceous mudstones, amber is very common and occurs randomly distributed as mm-sized droplets to dm-sized lumps and blocks, showing no signs of sorting and abrasion by reworking and transport. Combined with reconstructions from sedimentological and palynological data (Pocknall, 1982; Lindqvist, Gard & Lee, 2016), this indicates *in situ* resin deposition and amber formation in a domed forest swamp adjacent to a brackish mire or saltmarsh within an estuarine paleoenvironment. A comprehensive facies analysis of Pomahaka Formation was published by Lindqvist, Gard & Lee (2016). The late Oligocene age (Chattian, New Zealand stage Duntroonian, 27.3–25.3 Ma) for the Pomahaka Formation has been established on palynomorph and molluscan biostratigraphy (Wood, 1956; Pocknall, 1982; Beu & Maxwell, 1990).

## Materials and Methods

### Material

Thirteen Chironomidae inclusions from Pomahaka amber were studied herein, including four specimens reported as *Bryophaenocladius* Thienemann, 1934 or closely related to it by Schmidt et al. (2018) and nine newly discovered specimens. Three of the *Bryophaenocladius* specimens reported by Schmidt et al. (2018) are fossilized in a single piece of amber and a further specimen in a separate piece. The collection number No. OU35028.2 collectively assigned to all four specimens in Schmidt et al. (2018) is here replaced by individual numbers for each specimen (Nos. OU47579–OU47582). The nine new specimens originate from a single amber piece made up of multiple thin layers formed by successive resin flows but were separated and prepared as individual pieces (Figs. 1B, 1C). The type material and associated specimens are deposited in the Geology Museum of the Geology Department, University of Otago (OU); collection numbers are provided below in the Systematic Paleontology section.

### Preparation and imaging

Layered pieces of amber were microscopically examined for biological inclusions and subsequently separated along surfaces of individual resin flows. In instances where this resulted in the exposure of wings at the surface of the amber piece, wings were photographed with a binocular stereomicroscope (Carl Zeiss Stemi 508 with a Canon EOS 70D digital camera) prior to further preparation. Where possible, the thin and brittle, inclusion-bearing amber shards were ground and polished to obtain dorsal, ventral and/or lateral views of inclusions. Polished amber shards, and those too small and fragile for polishing, were embedded in epoxy resin to stabilise specimens, applying the protocol provided by Sadowski et al. (2021). Epoxy-embedded amber pieces were ground and polished using a grinder/polisher machine (Buehler Eco-Met 250) and CarbiMet silicon carbide abrasive papers (CarbiMet) and/or manually using a set of wet silicon carbide abrasive papers (FEPA P #220–4000).

Specimens were studied with a Carl Zeiss AxioScope A1 compound microscope and photographed with a Canon 5D digital camera. Figures were generated with Helicon Focus (8.2.0) software and enhanced using Adobe® Photoshop CC. Line drawings were prepared with Inkscape 1.1 software.

### µCT-scanning

Two Chironomid specimens preserved in close proximity in nearly opaque amber could not be separated and studied by light microscopy. These specimens (No. OU47580, No. OU47581) were scanned on the Imaging Beamline P05 (Lytaev et al., 2014) operated by the Helmholtz-Zentrum Hereon at the PETRA III storage ring (Deutsches Elektronen Synchrotron—DESY, Hamburg, Germany), using a photon energy of 18 keV and a sample-to-detector distance of 100 mm. Projections were recorded with a custom 20 MP CMOS imaging system with an effective pixel size of 1.28 µm (Lytaev et al., 2014). For each tomographic scan, 3,601 projections were recorded at equal intervals between 0 and π. Reconstruction was carried out by applying a transport of intensity phase retrieval approach and using the filtered back projection algorithm (FBP). This workflow was carried out in a custom reconstruction pipeline using MATLAB (Math-Works) and the Astra Toolbox (Moosmann et al., 2014; Van Aarle et al., 2015, 2016). Raw projections were binned twice for further processing, resulting in an effective pixel size of the reconstructed volume (voxel) of 2.56 µm. Scanned volumes were reconstructed using Drishti ver. 2.6.6 (Limaye, 2012). To decrease the demands for computer memory, we converted all stacks into eight-bit tiffs, downscaled all tiffs by 50% and subsequently cropped the empty space around the amber piece using Fiji ‘scale’ and ‘crop’ functions (Schindelin et al., 2012). Volumes were rendered in Drishti ver. 2.6.6 (Limaye, 2012).

### Terminology and taxonomy

Our morphological terminology is based on Sæther (1980) and Marshall et al. (2017). Specimens were evaluated using the keys provided by Freeman (1959), Sæther (1973, 1977, 1983), Albu (1974), Sublette & Wirth (1980), Pinder & Armitage (1986), Armitage (1987), Cranston, Oliver & Sæther (1989), Willassen (1996), Andersen & Schnell (2000), Wang, Sæther & Andersen (2001), Kaczorowska & Giłka (2002), Wang, Liu & Epler (2004), Makarchenko & Makarchenko (2006), Wang, Andersen & Sæther (2006), Langton & Pinder (2007), Du, Wang & Sæther (2011), Hazra & Das (2011), Epler (2012), Lin, Qi & Wang (2012), Moubayed & Lods-Crozet (2022), and Moubayed & Langton (2023).

Leg measurements of specimens are mainly approximated values only, due to the difficulty of measuring the variously oriented legs in the amber.

The electronic version of this article in Portable Document Format (PDF) will represent a published work according to the International Commission on Zoological Nomenclature (ICZN), and hence the new names contained in the electronic version are effectively published under that Code from the electronic edition alone. This published work and the nomenclatural acts it contains have been registered in ZooBank, the online registration system for the ICZN. The ZooBank LSIDs (Life Science Identifiers) can be resolved and the associated information viewed through any standard web browser by appending the LSID to the prefix http://zoobank.org/. The LSID for this publication is: urn:lsid:zoobank.org:pub:1B39CC4B-AA24-4D9F-819B-F1834E615C5C. The online version of this work is archived and available from the following digital repositories: PeerJ, PubMed Central SCIE and CLOCKSS.

## Results

### Systematic Paleontology

Order **Diptera** Linnaeus, 1758

Family **Chironomidae** Newman, 1834

Subfamily **Orthocladiinae** Kieffer, 1911

Genus ***Bryophaenocladius*** Thienemann, 1934

**Figure 2:**
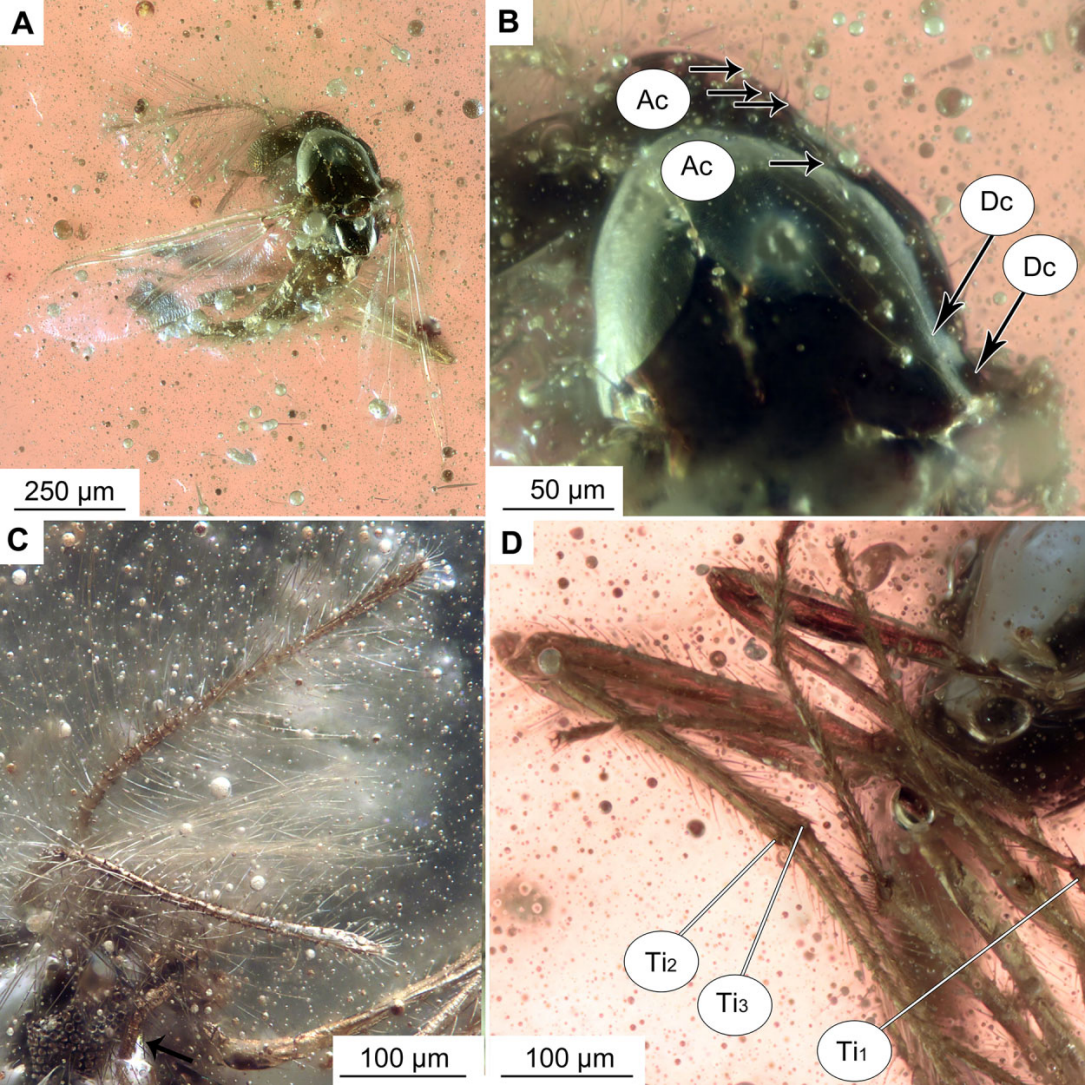
*Bryophaenocladius zealandiae* sp. nov. Baranov, holotype OU47576. (A) Habitus, dorsal view. (B) Habitus, ventral view. (C) Antenna, ventral view. (D) Tibial spurs. Abbreviations: Ac, Acrostichal setae; Dc, dorsocentral setae; Ti_1_, foreleg tibia; Ti_2_, midleg tibia; Ti_3_, hindleg tibia.

**Figure 3:**
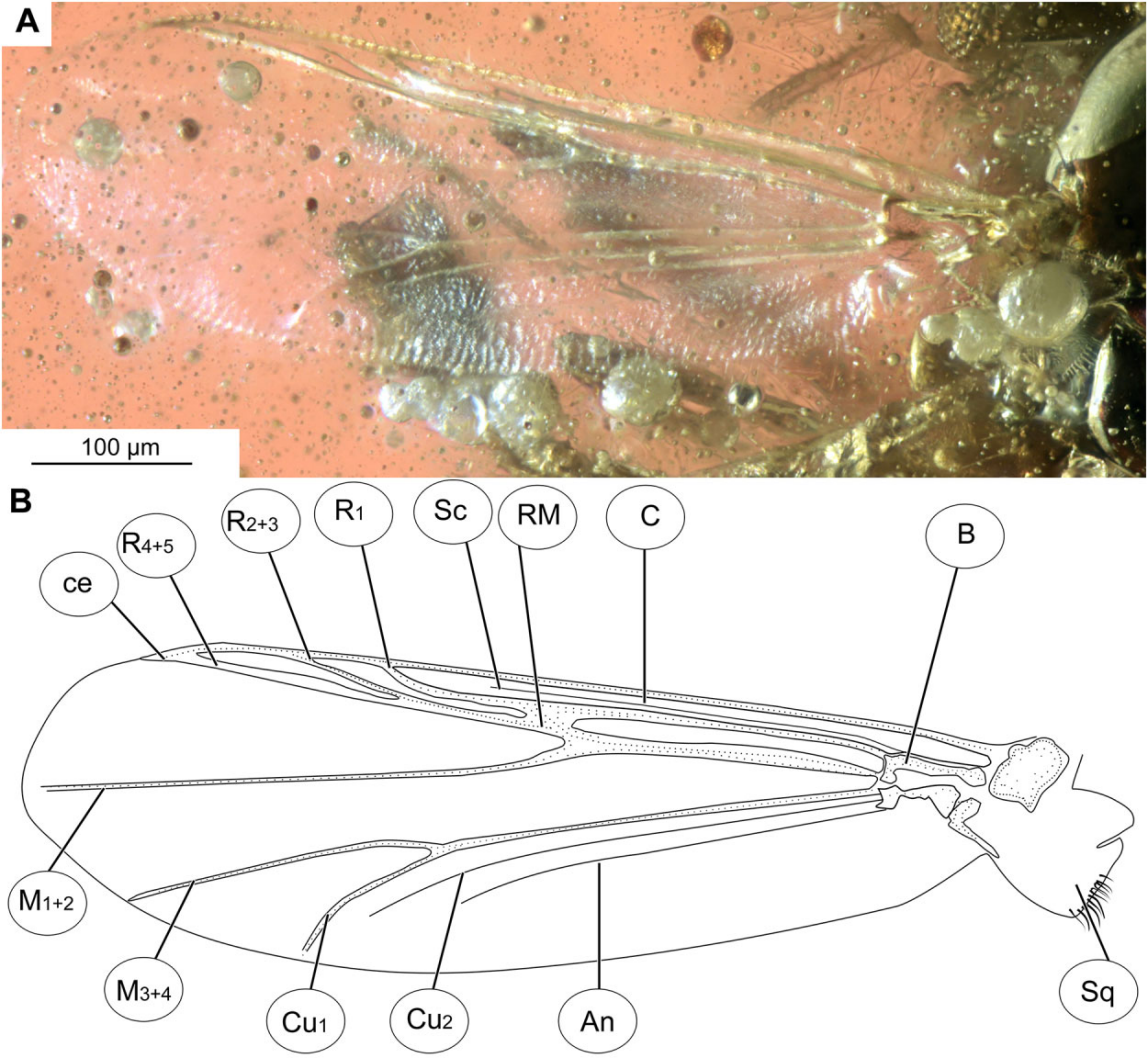
*Bryophaenocladius zealandiae* sp. nov. Baranov, wing of holotype OU47576. (A) Photomicrograph. (B) Line drawing. Abbreviations: An, anal vein; B, brachiolum; C, costal vein; ce, costal extension; Cu_1_, cubital vein 1; Cu_2_, cubital vein 2; M_1+2_, medial vein 1+2; M_3+4_, medial vein 3+4; R_1_, radial vein 1; R_2+3_, radial vein 2+3; R_4+5_, radial vein 4+5; RM, radial medial crossvein; Sc, subcostal vein; Sq, squama.

**Figure 4:**
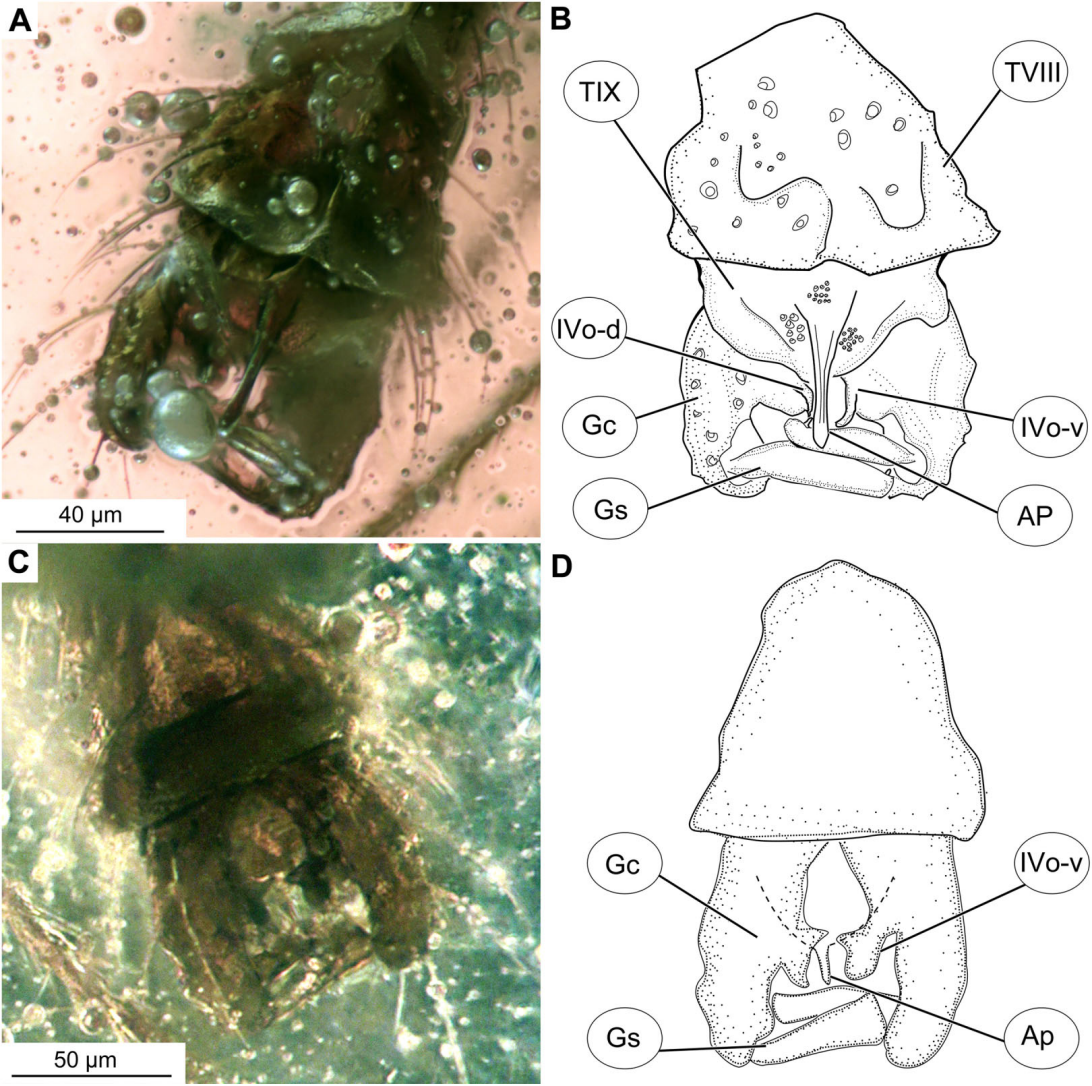
*Bryophaenocladius zealandiae* sp. nov. Baranov, hypopigium of holotype OU47576. (A) Photomicrograph, dorsal. (B) Line drawing, dorsal. (C) Photomicrograph, ventral. (D) Line drawing, ventral. Abbreviations: AP, anal point; Gc, gonocoxite; Gs, gonostylus; IVo, inferior volsella; TVIII, abdominal tergite 8; TIX abdominal tergite 9.

**Figure 5:**
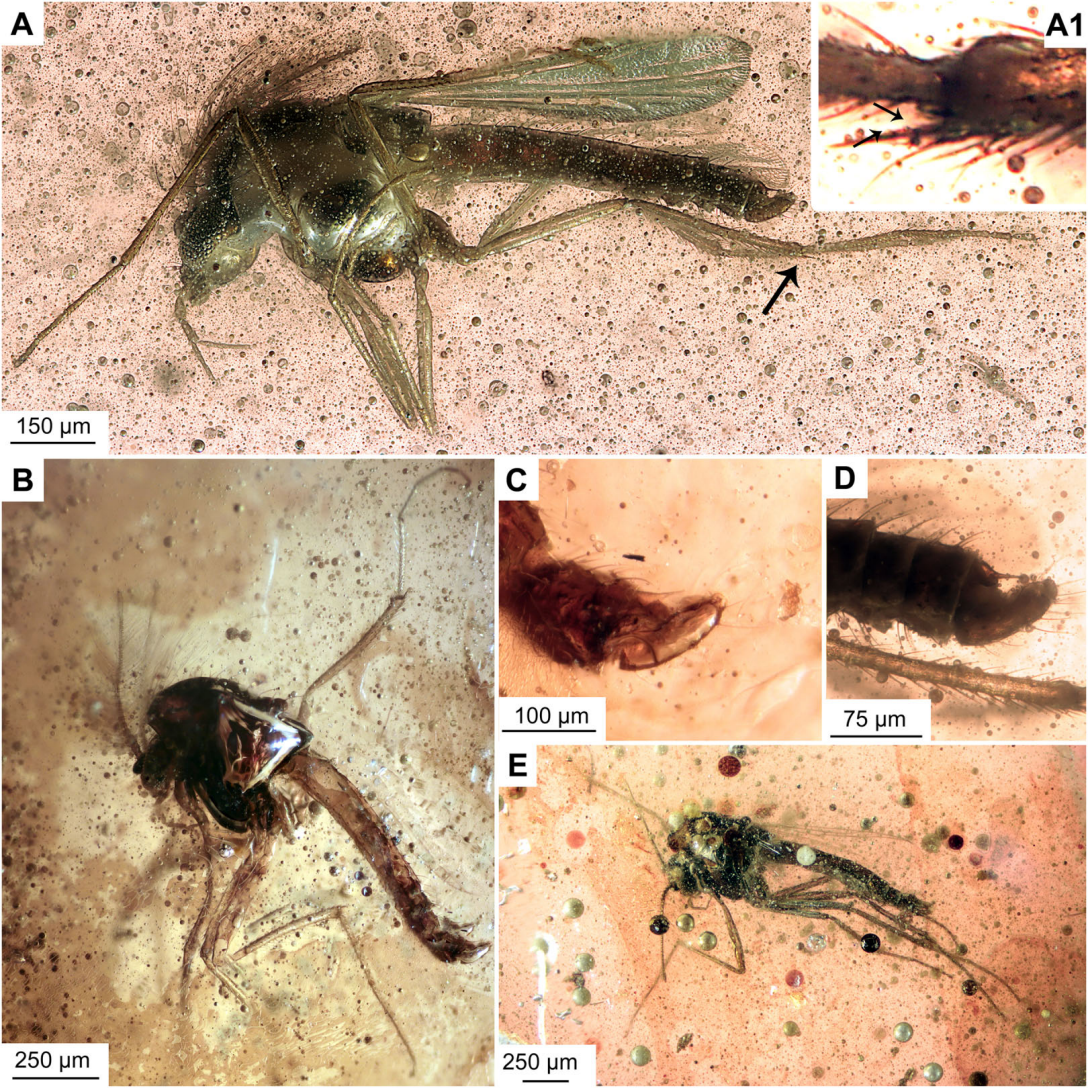
*Bryophaenocladius zealandiae* sp. nov. Baranov, paratypes. (A, B) Habitus and hypopygium of paratype OU47540. (A1) Hindtibia spurs. (C, D) Hypopygium and habitus of paratype OU47575. (E) Habitus of paratype OU47572.

**Figure 6:**
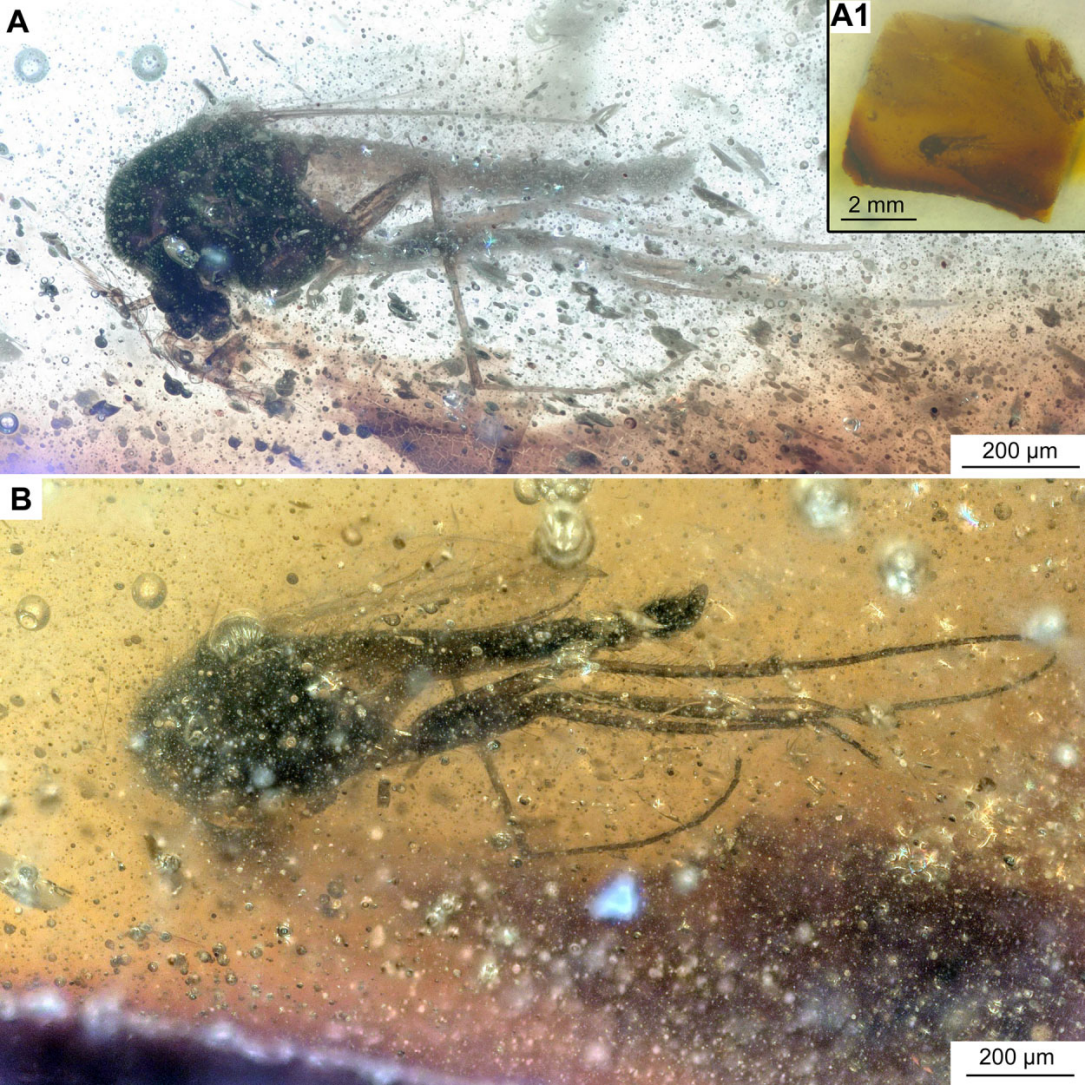
*Bryophaenocladius zealandiae* sp. nov. Baranov, associated specimen OU47579. (A) Habitus. (A1) Overview of the amber piece containing the specimen. (B) Habitus, opposite side of the body.

**Figure 7:**
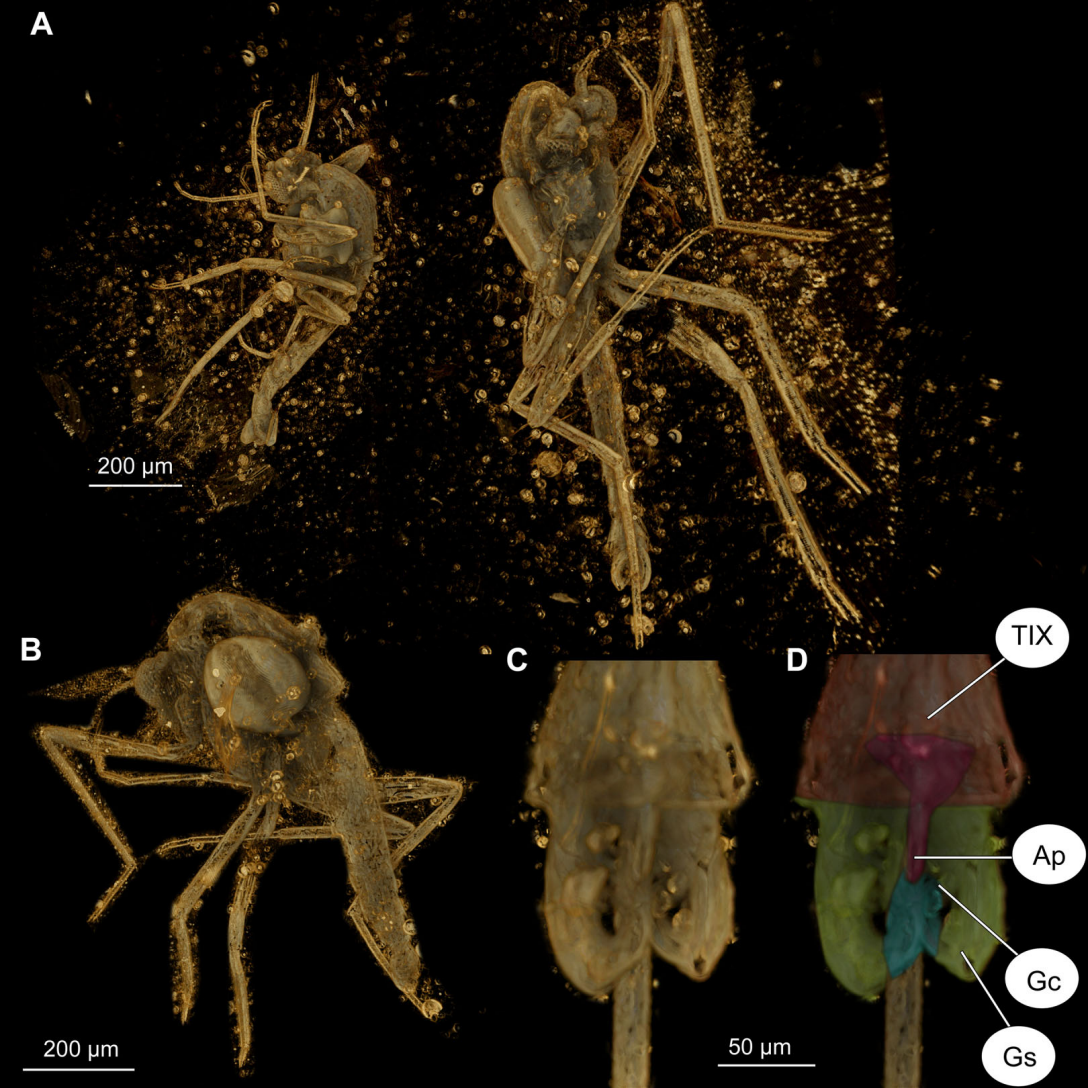
*Bryophaenocladius zealandiae* sp. nov. Baranov, CT scans of associated specimens. (A) Habitus of specimens OU47580 and OU47581 in the same amber piece. (B) Habitus of specimen OU47580, dorso-lateral view. (C) Hypopygium of specimen OU47581. (D) Hypopygium (OU47581), marked. Abbreviations: AP, anal point; Gc, gonocoxite; Gs, gonostylus; TIX, abdominal tergite 9. (E) Overview of the amber piece containing specimens OU47580, OU47581 and OU47582.

***Bryophaenocladius zealandiae* sp. nov. Baranov**

(Figs. 1C, 2–7; Table 1)

**Table 1:** Length (in μm) of leg segments of *Bryophaenocladius zealandiae* sp. nov. Baranov, males (measured on different numbers of specimens, depending on the preservation of the leg segments of the fossil).

Leg	Femora	Tibia	Ta_1_	Ta_2_	Ta_3_	Ta_4_	Ta_5_
Foreleg	255–547	255–570	136–260	58–100	50–84	36–45	50–65
	350 (*n* = 5)	370 (*n* = 5)	206 (*n* = 3)	78 (*n* = 3)	67 (*n* = 3)	41 (*n* = 3)	55 (*n* = 3)
Midleg	255–543	248–656	136–287	66–145	67–82	57–88	44–84
	400 (*n* = 6)	374 (*n* = 4)	202 (*n* = 3)	96 (*n* = 3)	76 (*n* = 3)	70 (*n* = 3)	63 (*n* = 3)
Hindleg	305–541	290–607	202–459	81–177	85–111	45–73	43–60
	419 (*n* = 6)	454 (*n* = 6)	286 (*n* = 6)	131 (*n* = 5)	100 (*n* = 5)	57 (*n* = 5)	52 (*n* = 4)

**Note:**

Values are given as min–max range and mean.

**Zoobank LSID:** urn:lsid:zoobank.org:act:2FE3D4EA-5F0B-4BB4-A2CC-E88557CE825

**Holotype.** No. OU47576, adult male, complete specimen in a piece of translucent, yellowish-orange amber with dimensions of 8 × 4 × 0.5 mm. Head and thorax covered by cloudy coating ventrally and dorsally, and parts of the thorax and abdomen obscured by numerous bubbles (Figs. 1C, 2–4).

**Paratypes.** No. OU47540, No. OU47572 and No. OU47575, adult males, generally well preserved but some morphology obscured by air bubbles (Fig. 5)

**Associated specimens.** No. OU47579 in a semi-translucent piece, No. OU47580, No. OU47581 and No. OU47582 together in one nearly opaque piece, mostly obscured by detritus and air bubbles; all adult males (Figs. 6, 7).

**Derivation of name.** The specific epithet refers to the largely submerged continent Zealandia.

**Type locality and horizon.** Temporary lignite pit, site G45/f0107, near Tapanui, southern New Zealand; Pomahaka Formation, late Oligocene (Chattian, New Zealand stage Duntroonian).

**Diagnosis.** The new species can be easily distinguished from any living and fossil *Bryophaenocladius* species based on the combination of the midlegs without tibial comb and with a reduced tibial spur, tapering anal point, and bi-lobed inferior volsella, together with gonostylus with a gentle curve, with a tip directed postero-laterally.

**Description**

**Habitus:** Total length 1.2–1.7 mm. Overall light yellowish-brown coloration, with thorax and pedicelli darker than the rest of the body.

**Head:** Eyes bare, kidney-shaped, without dorsomedial extension. Palpomeres (2–5) length in µm (*n* = 2, No. OU47572, No. OU47575): 23, 48–60, 80, 92–93 (Fig. 5A). Clypeus square, with at least eight setae (paratype No. OU47575). Palpomere three with a possible small distal protrusion, but condition of specimens not permitting corroboration of that. Antennae with 13 flagellomeres, (flagellomeres measurable on holotype only, length in μm): Fm_1_: 14, Fm_2_: 16, Fm_3_: 25, Fm_4_: 25, Fm_5_: 19, Fm_6_: 21, Fm_7_: 21, Fm_8_: 17, Fm_9_: 15, Fm_10_: 24, Fm_11_: 15, Fm_12_: 23, Fm_13_: 166, AR = 0.7.

**Thorax:** Acrostichals setae 5–8, strong and decumbent, starting close to the antepronotum, getting larger towards the posterior. In holotype No. OU47576: 5 visible, paratype No. OU47540: 8 visible. Dorsocentrals 7, uniserial; scutellars 8, uniserial. Postnotum bare.

**Legs:** Leg segments lengths as listed in Table 1. Terminal tarsomeres without pulvilli, shape of all flagellomeres cylindrical. Foreleg tibial spurs 10–31 μm (*n* = 2), midlegs without tibial comb and with a reduced tibial spur (Fig. 2D), hindtibia with two spurs, short 6–16 μm (*n* = 3), long 17–31 μm (*n* = 3) hindtibia comb made of 7–9 (*n* = 3) setae. Spurs with very weak lateral denticles, compressed to the main spur’s shaft (Fig. 5A1). Tarsomeres without pseudospurs.

**Wings:** 0.71–0.96, mean = 0.83 mm long (*n* = 5). Anal lobe strongly reduced. Costal extension ca. 85 µm long (*n* = 1). Cu_1_ slightly sinuate. Squama fully fringed, with at least 11 setae (*n* = 1, holotype) (Figs. 3A, 3B). Wing membranes without macrotrichia, with coarse punctuation. Venation as in Fig. 3A.

**Hypopygium:** Anal point long, expanding distally, bare, 23–40 μm long (*n* = 3), parallel-sided for the most of the length, widening distally. Gonocoxite 60–140 μm long (*n* = 3), with a large, bi-lobed inferior volsella, consisting of larger, anvil-shaped ventral lobe, and smaller, finger-like lobe, directed medio-posteriorly (Figs. 4A–4D, 7C, 7D). Gonostylus with a gentle curve, with a tip diverging outside (laterally from the body’s midline), 43 μm long (*n* = 1, holotype). Megasetae present, crista dorsalis absent (Figs. 4A–4D).

**Taxonomic notes**

The new species belongs to the genus *Bryophaenocladius* based on the combination of bare eyes, bare wings, fringed squama, lateral spines compressed to the shaft of the tibial spurs (note that spines are very weak, but not dissimilar from *B. muscicola* Kieffer, 1906 or *B. chrissichuckorum* Epler, 2012), pulvilli absent, acrostichal setae strong and decumbent, comb present on the hindtibia, and anal point well developed (Cranston, Oliver & Sæther, 1989). In the absence of a modern, comprehensive revision of the genus *Bryophaenocladius* it is difficult to ascertain relations between the new fossil species and other species of *Bryophaenocladius*. The general shape of the hypopygium, particularly the long, distally expanding anal point, is highly reminiscent of *B. beuki*, Baranov, Andersen & Hagenlund, 2015 from Baltic amber. Among extant taxa, the hypopygium of the new species is quite similar to *B. psilacrus* Sæther, 1982 in the bi-lobed inferior volsella, with larger anvil-shaped ventral lobe and a smaller, finger-like dorsal lobe, the long anal point, and the gently curving gonostylus, without crista dorsalis, as well as to *B. vernalis* (Goetghebuer, 1921) (Brundin, 1956; Makarchenko & Makarchenko, 2006).

**Figure 8:**
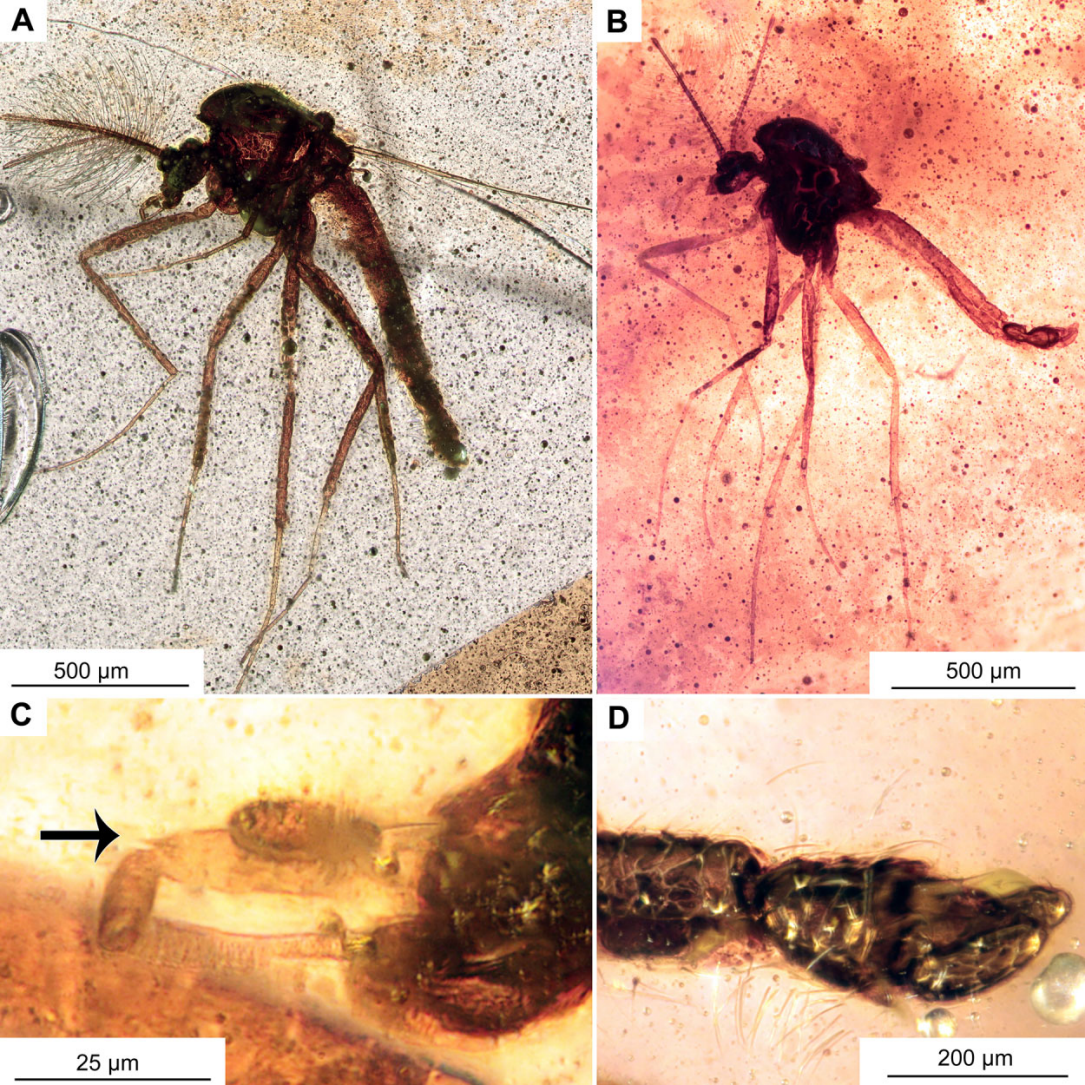
Morphotype 1 cf. *Bryophaenocladius zealandiae*. (A) Habitus of specimen OU47574. (B) Habitus of specimen OU47573. (C) Palpomere 3 (OU47574), arrow marks distal protrusion. (D) Hypopygium, lateral view (OU47574).

**Morphotype 1 cf. *Bryophaenocladius zealandiae***

(Figs. 1C, 8, Table 2)

**Table 2:** Length (in μm) of leg segments of morphotype 1 cf. *Bryophaenocladius zealandiae* (measured on two specimens).

Leg	Femora	Tibia	Ta_1_	Ta_2_	Ta_3_	Ta_4_	Ta_5_
Foreleg	471–518	461–606	325–415	111–115	101–109	72–136	46–79
Midleg	409–511	471–561	178–250	93–144	65–117	33–49	50–51
Hindleg	489–565	530–570	293–340	153–189	95–142	52–79	62–72

**Note:**

Values are given as min–max range.

**Material.** No. OU47573 and No. OU47574, both complete and fairly well visible within yellowish-orange translucent amber.

**Description**

**Habitus:** Total length 2 mm, wing length 1 mm (*n* = 2). Colour: dark brown head and body, and legs of a lighter-brown colour.

**Head:** Eyes bare, kidney-shaped, without dorsomedial extension. Palpomeres (2–5) length in µm (*n* = 2): 26, 45–77, 46–52, 83–100 (Figs. 7A–7D). Clypeus square. Palpomere three with a distinct conical protrusion on the distal end.

**Thorax:** Acrostichals setae strong and decumbent, 8 present (*n* = 2). Dorsocentrals present but difficult to count, uniserial, at least 4. Postnotum bare. Anepisternum and epimeron without leaf-shaped setae.

**Legs:** Leg segments lengths as listed in Table 2. Terminal tarsomeres without pulvilli, shape of all the flagellomeres cylindrical. Foreleg tibial spurs 16 μm (*n* = 2), midlegs without tibial spur, hindtibia with two spurs, short 27 μm (*n* = 1), long 30–46 μm (*n* = 2) hindtibia comb made of 6–8 (*n* = 2) setae. Tarsomeres without pseudospurs. Lateral spines compressed to the shaft of the tibial spurs.

**Wings:** 1 mm long (*n* = 2). Details of venation not observable.

**Hypopygium:** Only visible in lateral aspect. Anal point bare, ca 50 μm long (*n* = 1). Gonocoxite ca. 150 μm long (*n* = 1).

**Taxonomic notes**

This morphotype is similar to *Bryophaenocladius zealandiae* sp. nov.; however, wing venation and structure of terminalia are not decipherable. Should they indeed be members of *Bryophaenocladius zealandiae* sp. nov., this will corroborate that this species has a distal projection on the end of palpomer 3, supporting affinity with the subgenus *Odontocladius* Albu, 1974.

Genus ***Pterosis*** Sublette & Wirth, 1980

**Amended generic diagnosis:** Can be distinguished from any other Chironomidae based on the combination of absent apical setae on the last flagellomere of a male, the well-developed antepronotum, scutellars biserial, wing membrane fully covered with macrotrichia, R_2+3_ vein reaching almost to the apex of the R_1_ vein, squama fringed, inferior volsella with various density of setae, anal point heavily setose, gonostylus with a crista dorsalis of variable size.

**Figure 9:**
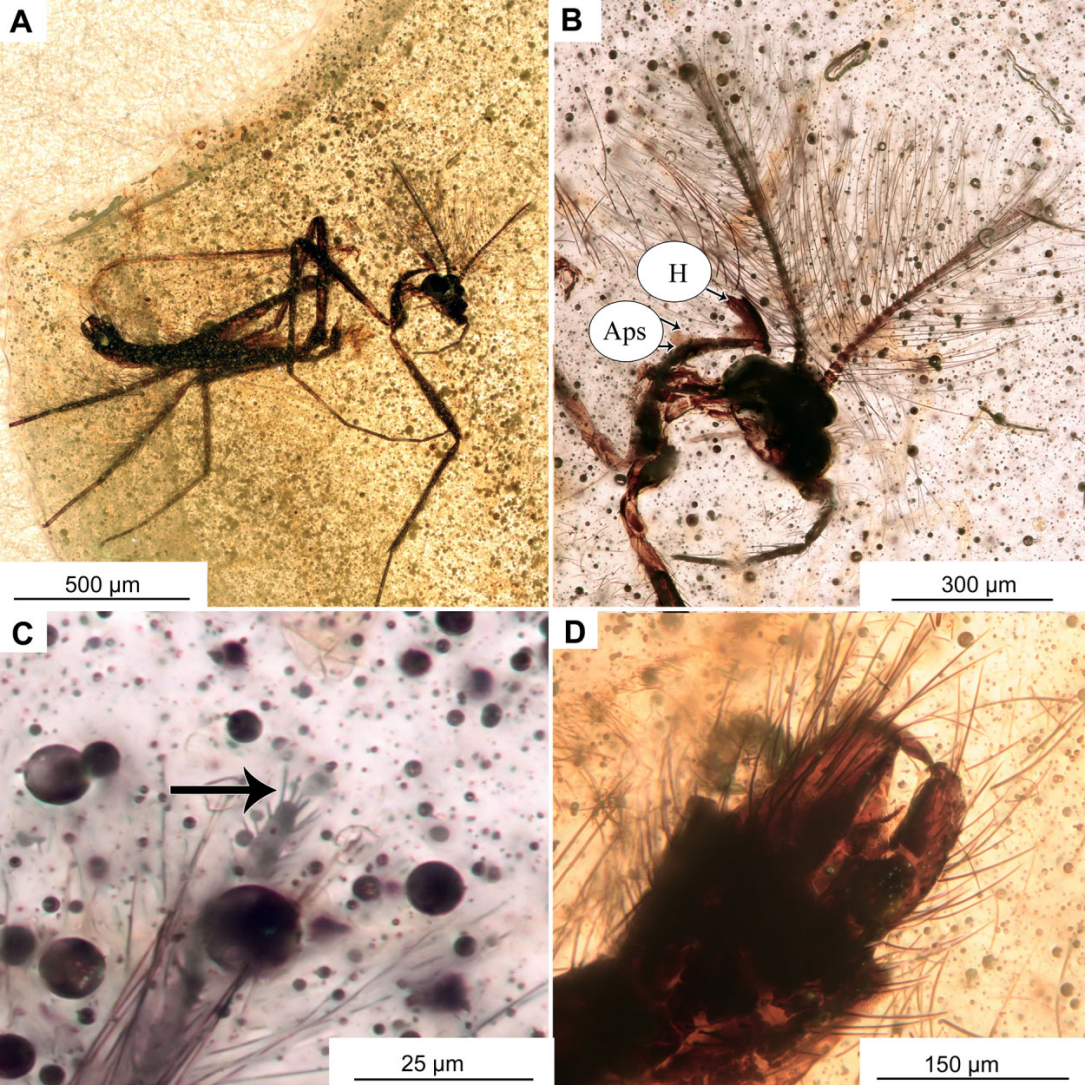
*Pterosis extinctus* sp. nov. Baranov, holotype OU47546, male. (A) Habitus. (B) Head. (C) Last flagellomere’s a crown of gentle sensillae marked by arrow. (D) hypopygium , ventral view. Abbreviations: Aps, antepronotal setae; H, humeral setae.

**Figure 10:**
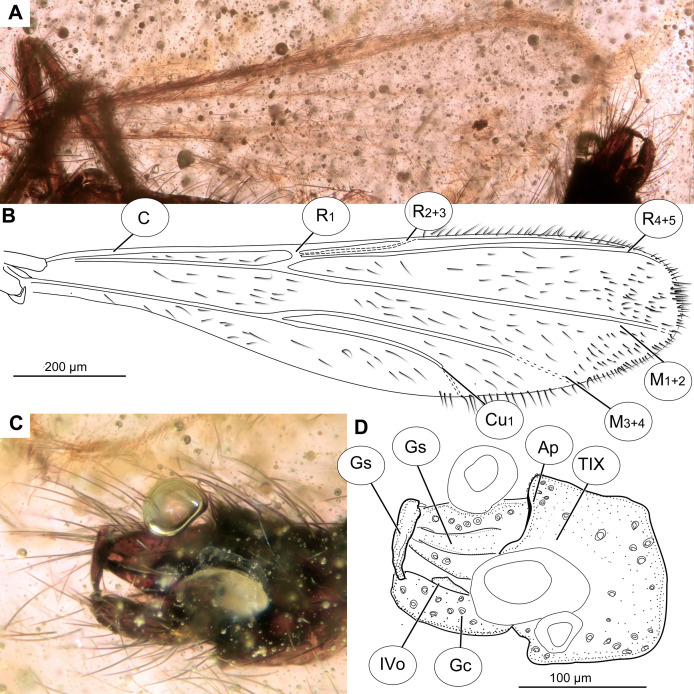
*Pterosis extinctu*s sp. nov. Baranov, holotype OU47546. (A) Photomicrograph of wing. (B) Line drawing of wing. (C) Photomicrograph of hypopygium, dorsal view. (D) Line drawing of hypopygium, dorsal view. Abbreviations: AP, anal point; Gc, gonocoxite; Gs, gonostylus; IVo, inferior volsella; TIX, abdominal tergite 9; C, costal vein; Cu_1_, cubital vein 1; M_1+2_, medial vein 1+2; M_3+4_, medial vein 3+4; R_1_, radial vein 1; R_2+3_, radial vein 2+3; R_4+5_, radial vein 4+5.

***Pterosis extinctus* sp. nov. Baranov**

(Figs. 1C, 9, 10, Table 3)

**Table 3:** Length (in μm) of leg segments of *Pterosis extinctus* sp. nov. Baranov, male holotype No. OU47546.

Leg	Femora	Tibia	Ta_1_	Ta_2_	Ta_3_	Ta_4_	Ta_5_
Foreleg	704	741	423	175	222	161	217
Midleg	596	520	468	124	76	–	–
Hindleg	615	783	–	–	–	–	–

**Zoobank LSID:** urn:lsid:zoobank.org:act:DBC38BCD-7C3A-489A-8B47-344576745488

**Holotype.** No. OU47546; male, partially preserved (thorax is missing), in a piece of semi-translucent, yellow amber (7 × 5 × 0.5 mm) with abundant small air-bubbles (Figs. 1C, 9, 10).

**Derivation of name.** After Latin “extinctus”, meaning extinct.

**Type locality and horizon.** Temporary lignite pit, site G45/f0107, near Tapanui, southern New Zealand; Pomahaka Formation, late Oligocene (Chattian, New Zealand stage Duntroonian).

**Diagnosis.** This fossil species can be distinguished from the only other known *Pterosis* species, *Pterosis wisei* Sublette & Wirth, 1980, based on the combination of the following characters: apical flagellomere with a crown of sensillae, antepronotal setae present close to the midlength of the antepronotum, Cu_1_ slightly sinuate, inferior volsella lightly setose, anal point blunt and setose, gonostylus with weak crista dorsalis.

**Description**

**Adult male** (No. OU47546)

**Habitus:** Total length 2.3 mm. Colour: dark brown across the parts of the body.

**Head:** Eyes bare, presence of the dorsomedial extension impossible to ascertain. Palpomeres (3–5) length in µm (*n* = 1): 115, 126, 143 (Fig. 8B). Clypeus square with at least 10 setae. Antennae with 13 flagellomeres, (flagellomeres measurable on holotype only, length in μm): Fm_1_: 27, Fm_2_: 30, Fm_3_: 19, Fm_4_: 31, Fm_5_: 30, Fm_6_: 31, Fm_7_: 25, Fm_8_: 30, Fm_9_: 27, Fm_10_: 28, Fm_11_: 28, Fm_12_: 31, Fm_13_: 360, AR = 1.1 Flagellomere 13 with a crown of gentle sensillae (Fig. 9C).

**Thorax:** Most of the thorax, except for antepronotum and part of the scutum, missing. Antepronotal lobes well developed, meeting medially. Strong antepronotal setae present (at least two), reaching the mid-length of the antepronotal lobes. Small piece of mesonotum still preserved (Fig. 10A), strongly projecting forward, over the head. Three humerals visible (Fig. 9B).

**Legs:** Leg segment lengths as listed in Table 3. Foreleg tibial spurs 30 μm (*n* = 1), presence and number of other spurs impossible to ascertain. Presence of the pseudospurs on the foreleg tibia impossible to ascertain, due to it being surrounded by a dense cloud of bubbles. Pulvilli absent.

**Wings:** 1.5 mm long (*n* = 1). Wing membrane densely covered with macrotrichia. Cu_1_ slightly sinuate, costal extension produced slightly beyond R4+5 insertion, otherwise, venation as in Fig. 10B. Squama invisible (Figs. 10A, 10B).

**Hypopygium:** With numerous long setae, gonocoxite 114 μm long (*n* = 1). Gonostylus ca. 70 μm long (*n* = 1), expanding distally, without obvious crista, but with sub-oval expansion ventrally, megasetae short and sturdy. Anal point short, cresting top of tergite IX, with several long setae (Figs. 10C, 10D). Inferior volsella subrectangular, narrowing distally (Figs. 10C, 10D). Presence of virga impossible to ascertain, but since hypopygium is partially transparent, we can rule out presence of extremely strong and sclerotized virga.

**Taxonomic notes**

This species is attributed to the genus *Pterosis* based on the combination of bare eyes, apical flagellomere without subapical sensillae, wing fully covered with macrotrichia with costal extension, antepronotals present, mesonotum strongly projected forward over the head, humerals present, presence of crest-like anal point on tergite IX, absence of virga and overall extremely high density of setae on the body (Sublette & Wirth, 1980). Since the hypopygium and tergite VIII are partially translucent in the male specimen, and the extremely large and sclerotized virga is not visible, its presence is unlikely. Absence of the apical setae on the 13^th^ flagellomere differentiates this species from representatives of *Gymnometriocnemus* Edwards, 1932a (Sublette & Wirth, 1980; Sæther, 1983; Stur & Ekrem, 2015). The species can be differentiated from representatives of *Allometriocnemus* Freeman, 1961, by combination of the lobes of antepronotum meeting medially and wing membrane being completely covered with macrotrichia (Freeman, 1961; Sublette & Wirth, 1980).

*P. extinctus* can be easily differentiated from *P. wisei* by antepronotal setae of the former being closer to the midlength of the antepronotum, in contrast to *P. wisei*, whose antepronotals are all concentrated on the distal part of the antepronotum, as well as much smaller crista dorsalis of the new species (Sublette & Wirth, 1980).

**Figure 11:**
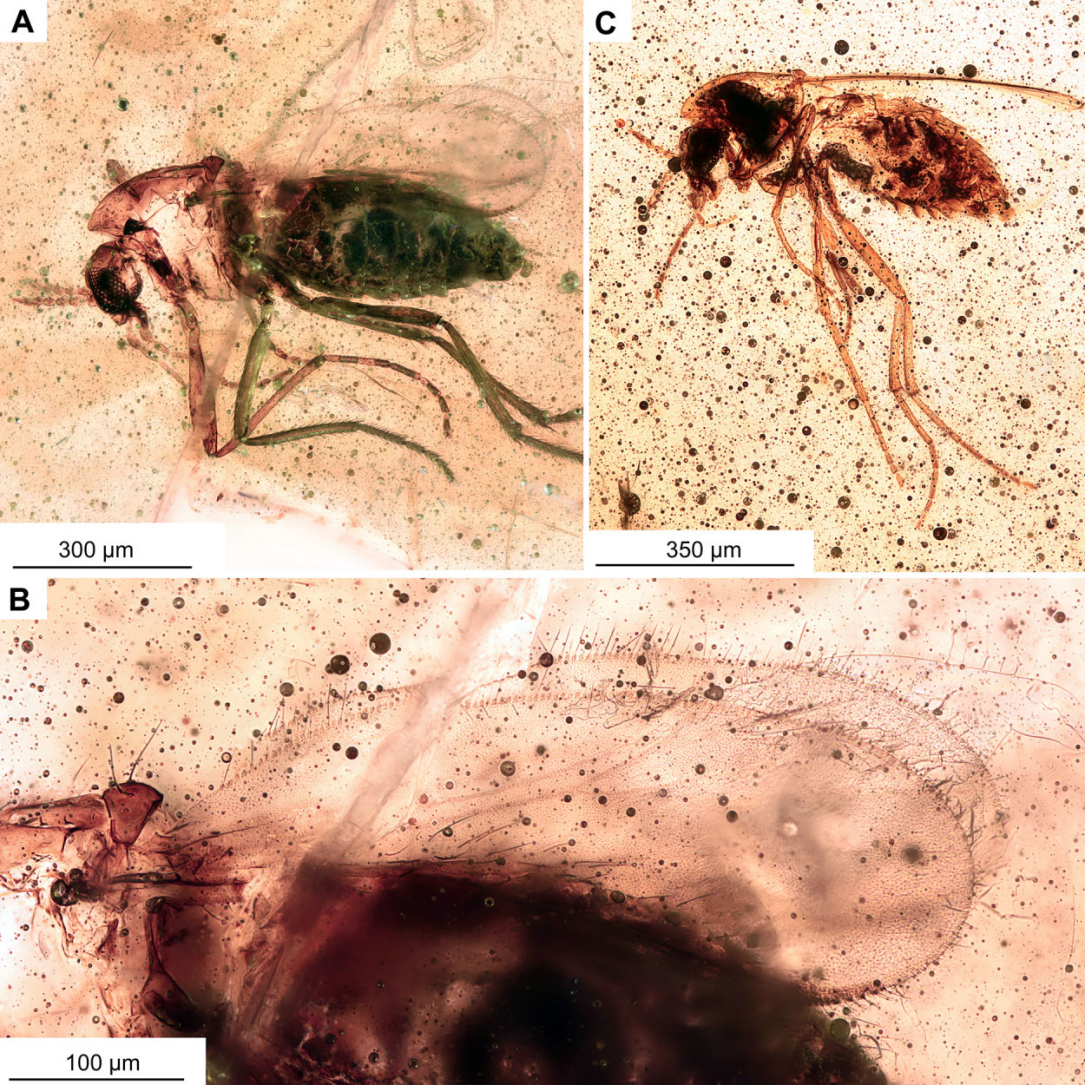
Morphotype 2, cf. Metriocnemini, females. (A, B) Habitus and wing of specimen OU47577. (C) Habitus of specimen OU47578.

**Figure 12:**
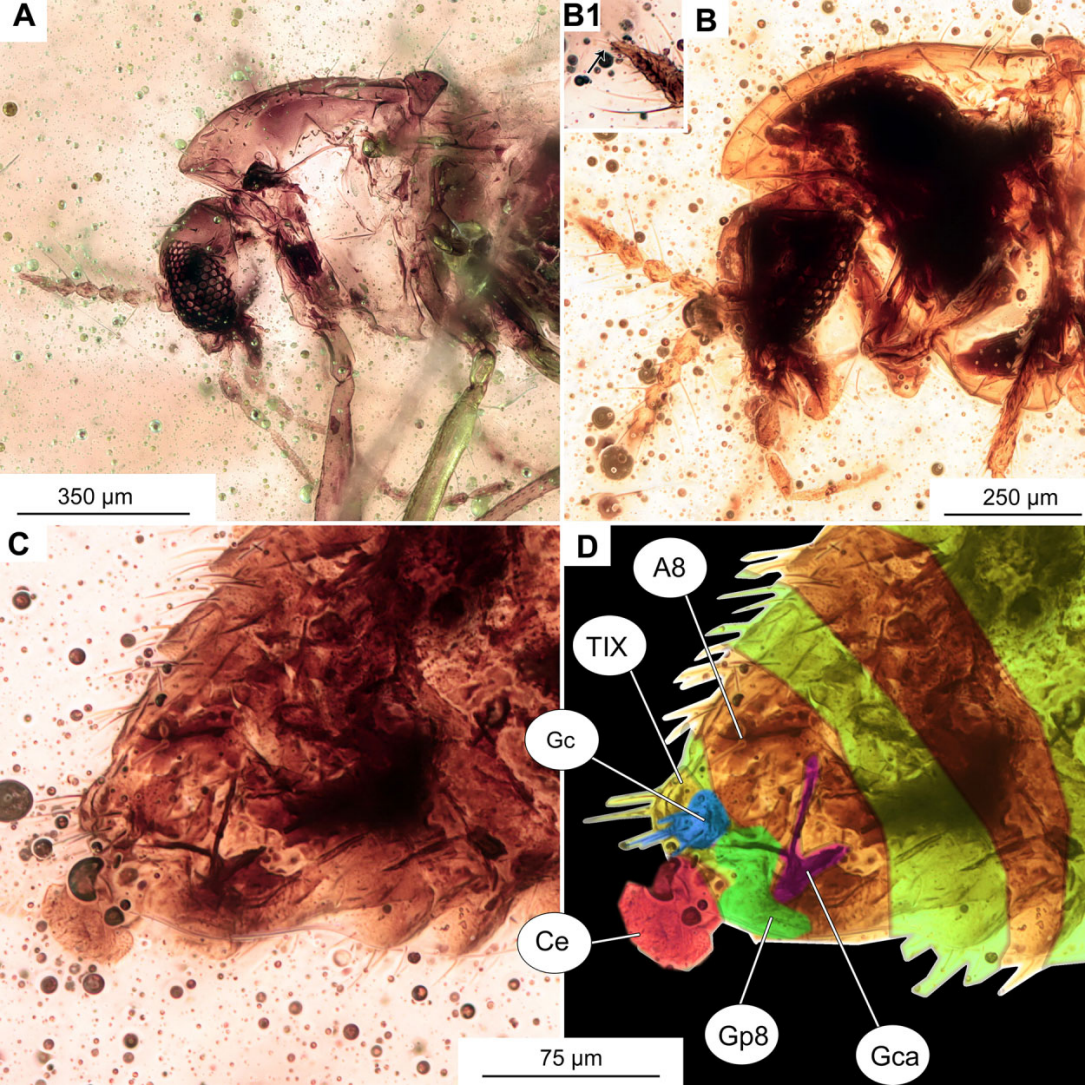
Morphotype 2, cf. Metriocnemini, females. (A) Head of specimen OU47577. (B) Head of specimen OU47578; B1 close-up of last flagellomere with apical setae. (C) Female genitalia (OU47578). (D) Female genitalia, marked (OU47578). Abbreviations: A8, abdominal segment 8; Ce, cerci; Gca, gonocoxite apodem; Gc, gonocoxite (8); Gp8, gonapophysis 8; TIX, tergite 9.

**Morphotype 2 cf. Metriocnemini**

(Figs. 11, 12, Table 4)

**Table 4:** Length (in μm) of leg segments of Morphotype 2, females (measured on different numbers of specimens, depending on the preservation of leg segments).

Leg	Femora	Tibia	Ta_1_	Ta_2_	Ta_3_	Ta_4_	Ta_5_
Foreleg	312	308	109–139	56–70	48–50	28–34	26–46
Midleg	238–282	259–266	92–100	39–49	33–34	23–29	38–47
Hindleg	267–269	211–296	145–171	55–69	70–89	37–40	42–50

**Note:**

Values are given as min–max range.

**Material.** No. OU47577 and No. OU47578, adult females, both complete and fairly well visible within yellowish-orange translucent amber.

**Description**

**Adult female**

**Habitus:** Total length 0.9–1.0 mm. Colour: dark brown across the parts of the body.

**Head:** Eyes bare, reniform. Palpomeres (2–5) length in µm (*n* = 2): 29 (*n* = 1, OU47578), 40–41, 41–42, 75–82 (Figs. 11A–11C, 12A, 12B). Clypeus square with at least 11 setae. Antennae with 5 flagellomeres, (*n* = 2, length in μm): Fl1: 76 (*n* = 1, OU47577), Fl2: 19–29, Fl3: 26–28, Fl4: 22–27, Fl5: 39–40. Flagellomere 5 with a weak but distinct subapical seta (Figs. 12A, 12B). Pedicellus cup-shaped.

**Thorax:** Acrostichals setae strong and decumbent, 5–8 (*n* = 2). Dorsocentrals biserial, upper row 5, lower row 8. Postnotum bare. Antepronotum 4. Anepisternum and epimeron without leaf-shaped setae. Prealars 3, humerals 4. Scuterals uniserial, 6.

**Legs:** Leg segments lengths as listed in Table 4. Foreleg tibial spurs 14–15 μm (*n* = 2), midtibial spur 11–15, hindtibial spur 26–37 (length in μm). Hindtibial comb made of 8–9 strong seate. Pulvilli absent, empodium feathery.

**Wing:** 0.63–0.72 mm long (*n* = 2). Wing membrane densely covered with macrotrichia. Cu_1_ slightly sinuate. Squama bare, costal extension pronounced (Fig. 11C). Wing with numerous macrotrichia, otherwise as shown on the Fig. 11B. Halters dark-brown in their entirety.

**Female genitalia:** Cerci very small, gonapophysis VIII divided into small mesal lobe and narrow dorsomesal lobe (Figs. 12C, 12D). Gonocoxite relatively small, with at least 5 strong setae. Tergite IX rounded, undivided (Figs. 12C, 12D).

**Taxonomic notes**

Dense macrotrichia of the wings, as well as dense setation of the thorax, with antepronotals present, is indicative of a close affinity of this morphotype with representatives of the genera *Metriocnemus* van der Wulp, 1874 or *Gymnometriocnemus*, with more precise determination of taxonomic affinity impossible without additional material (Sæther, 1977).

## Discussion

### Faunal affinities and biogeography

Chironomids have a long history, with the oldest representatives occurring in the uppermost Triassic of Europe (203 mya) (Krzemiński & Jarzembowski, 1999), although based on dated phylogenies the group is likely significantly older, at least 250 mya (Cranston, Hardy & Morse, 2012). The oldest Orthocladiinae fossils of *Lebanorthocladius furcatus* Veltz, Azar & Nel, 2007 are known from Lower Cretaceous Lebanese amber (Veltz, Azar & Nel, 2007). The long geological history and rich fossil record has made chironomids a suitable model group for historical biogeographic analyses. Following Hennig’s (1966) work on phylogenetic systematics, Lars Brundin became interested in applying principles of cladistic analysis and an emerging understanding of plate tectonics to the analysis of Chironomidae distribution in the Southern Hemisphere (Brundin, 1966). Brundin came to the conclusion that the majority of Chironomidae distribution patterns in Australia, Southern Neotropics and New Zealand can be explained by the break-up of Gondwana. Since then, however, our understanding of the assembly of New Zealand’s biota has become more refined. In particular the role of dispersal has become more widely accepted (*e.g*., Trewick, 2000; Sanmartin, Enghoff & Ronquist, 2001). The composition of the New Zealand Chironomidae, particularly the Orthocladiinae fauna, reflects a complex history influenced by both trans-Tasman and trans-Antarctic dispersal and vicariance following the break-up of Gondwana (Krosch & Cranston, 2013; Krosch et al., 2011, 2015).

*Bryophaenocladius* has a near worldwide distribution, although there are only preliminary records from Australia (Cranston, 1996) and the genus is seemingly absent from the extant fauna of New Zealand (Boothroyd & Forsyth, 2011; Ashe & O’Connor, 2012). This study follows on the specimens from Oligocene amber, preliminarily classified by us into genus *Bryophaenocladius* in our earlier article (Schmidt et al., 2018), which was the first record of *Bryophaenocladius* from New Zealand. We also noted that the BOLD V4 system has barcoding records of *Bryophaenocladius* in New Zealand (Schmidt et al., 2018) but, on closer examination, these belong to the two BOLD BINs BOLD:AAM6273 and BOLD:AAG1021. Representatives of these BINs all cluster around the Holarctic species *Bryophaenocladius ictericus* (Meigen, 1830). It is thus likely that this species has been historically introduced to New Zealand (and Australia) with agricultural produce, as *Bryophaenocladius* larvae are associated with agricultural plants (Cranston, 1987).

**Figure 13:**
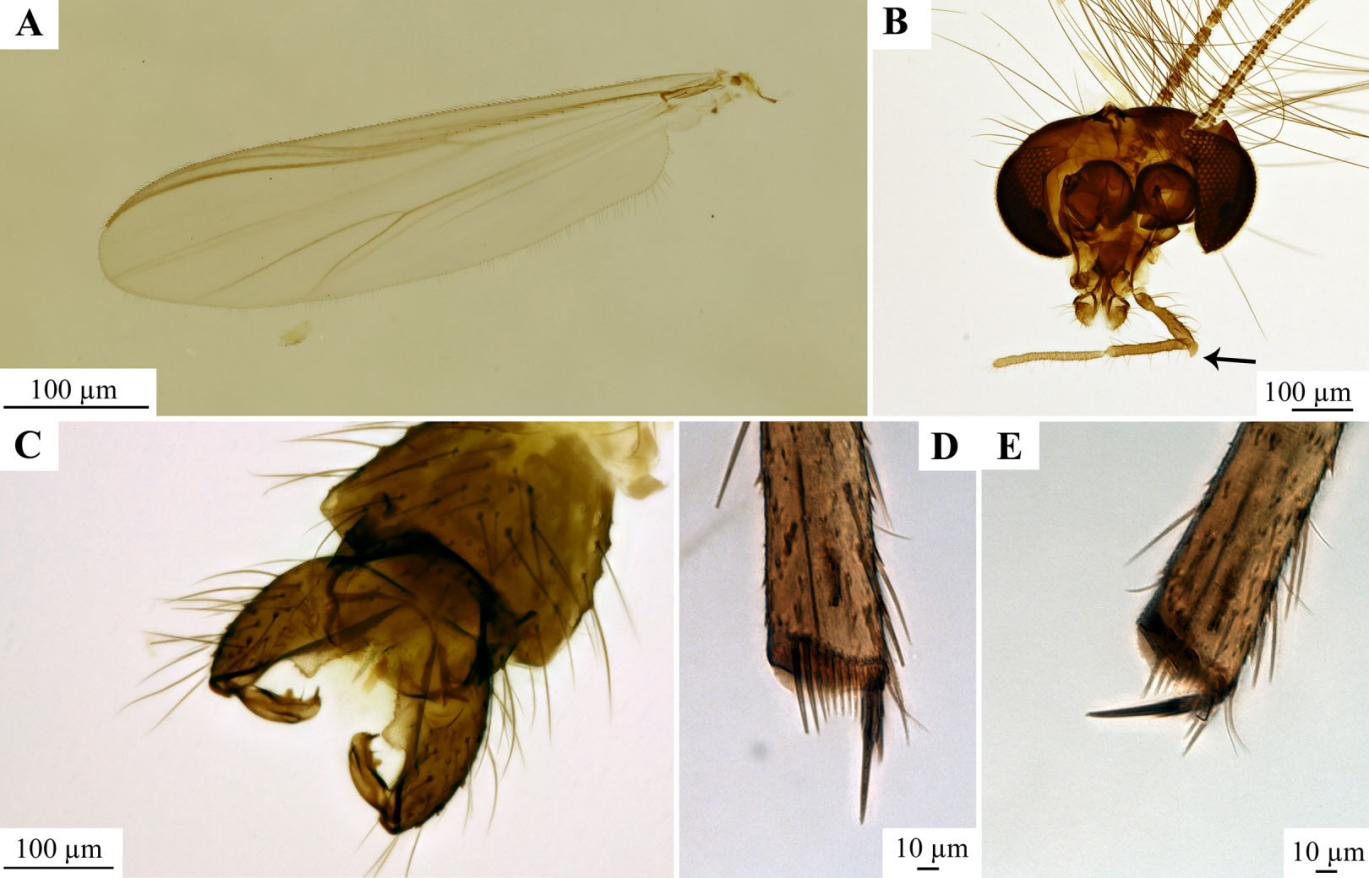
Holotype (adult male, number NZAC02044947) of *Kuschelius dentifer* Sublette & Wirth, 1980. (A) Wing. (B) Head, arrow marks an apical protrusion of the 3^rd^ palpomere. (C) hypopygium. (D) Midtibia with spurs and the comb. (E) Hindtibia with the spurs and comb. All photos in this plate are made by Dr. Leanne Elder, licensed under CC BY 4.0 and used with the photographer’s explicit permission.

While there appear to be no native species of *Bryophaenocladius* on the main islands of New Zealand, it is highly likely that the monotypic *Kuschelius dentifer* Sublette & Wirth, 1980, endemic to the sub-Antarctic Auckland Islands, is in fact a species of *Bryophaenocladius*. Sublette & Wirth (1980) erected the genus *Kuschelius* as intermediate between *Chaetocladius* Kieffer, 1911 and *Bryophaenocladius*, and distinguished *K. dentifer* from species of *Bryophaenocladius* by the presence of apical setae on the terminal flagellomere of the antenna and slightly diverted spines on the tibial spur of the hind leg (Figs. 13A–13E). However, these characters in combination with the structure of the hypopygium and the presence of the distal projection on the distal end of palpomere 3 fit well within the current definition of *Bryophaenocladius*, subgenus *Odontocladius* Albu, 1974 (Albu, 1974; Armitage, 1987; Moubayed & Langton, 2023). As pointed out by Sæther (1982) and Armitage (1987), *K. dentifer* is almost certainly a *Bryophaenocladius*, very similar to *B. brincki* (Freeman, 1955) originally described from South Africa. Molecular data on *K. dentifer* are not yet available. *B. zealandiae* sp. nov. Baranov from Pomahaka amber now confirms the occurrence of the genus *Bryophaenocladius* in Zealandia in the late Oligocene (~26 mya) and documents its post Oligocene extinction in New Zealand, at least on the main islands. The only two previously reported fossils of *Bryophaenocladius* (*B. beuki* Baranov, Andersen & Hagenlund, 2015 and *B. circumclusus* Seredszus & Wichard, 2007) are from Eocene Baltic amber. A review of modern *Bryophaenocladius* and additional fossils along with further fossil specimens would be of a great importance to deciphering the biogeographic history of this genus.

The genus *Pterosis* identified here from Pomahaka amber includes one extant species, *P. wisei* Sublette & Wirth, 1980, endemic to the subantarctic Auckland Islands and Campbell Island of New Zealand (Sublette & Wirth, 1980). The discovery of *Pterosis extinctus* sp. nov. Baranov in amber from the Pomahaka Formation shows the presence of *Pterosis* on mainland New Zealand in the late Oligocene.

### Paleoecology of non-biting midges from Pomahaka amber

It is notable that both newly discovered species of midges from Pomahaka amber belong to Chironomidae groups whose extant representatives have larvae that develop mostly in terrestrial and semi-aquatic habitats (Moller Pillot, 2013), and not in the aquatic habitat seen in larvae of most other chironomids. Larvae of *Bryophaenocladius* develop in wet mosses, decaying leaves or similar wet habitats (Strenzke, 1957; Moller Pillot, 2013). Larvae of *Pterosis* are unknown, but given the adults’ similarity to *Gymnometriocnemus* representatives, larvae of *Pterosis* likely develop in wet terrestrial habitats as well (Sublette & Wirth, 1980). Terrestrial and semi-terrestrial Chironomidae are relatively common in various amber deposits worldwide, probably due to their association with mosses and other microhabitats on the bark of the resin-producing trees or on the nearby forest floor (Solórzano-Kraemer et al., 2018). Prior to the research presented in this article, two fossil species of *Bryophaenocladius* were known: *B. beuki* Baranov, Andersen & Hagenlund, 2015 and *B. circumclusus* Seredszus & Wichard, 2007 and a probable larva of this genus (Baranov et al., 2019), all from Eocene Baltic amber. Until now no fossil *Pterosis* were known but there are numerous other fossils of Chironomidae whose extant representatives develop in terrestrial habitats, such as *Parametriocnemus* Goetghebuer, 1932, *Paraphaenocladius* Spärck & Thienemann, 1924, *Pseudorthocladius* Goetghebuer, 1943, *Smittia* Holmgren, 1869 and *Pseudosmittia* Edwards, 1932b (Zelentsov et al., 2012; Baranov, Andersen & Hagenlund, 2015). The prevalence of the Chironomidae with terrestrial larvae in certain amber deposits indicates high humidity in amber forest habitats, as relatively high and constant humidity is required by these groups of Chironomidae to finish larval development (Strenzke, 1957; Armitage, Cranston & Pinder, 1995, Zelentsov et al., 2012). The finding of terrestrial or semi-aquatic midges in Pomahaka amber is consistent with the paleo-environmental reconstruction. The amber-bearing lignites of Pomahaka Formation formed by *in-situ* growth and decomposition of wetland forest trees and litter in domed forest swamps (Lindqvist, Gard & Lee, 2016) and the palynomorph assemblage from the lignites includes ferns, shrubs, herbs and reeds associated with moist and damp habitats which indicates high humidity and high rainfall throughout the year (Pocknall, 1982).

## Additional Information and Declarations

### Data Availability

The following information was supplied regarding data availability:

All specimens included in this work are housed at the Geology Museum of the Geology Department, University of Otago (OU): *Bryophaenocladius zealandiae* sp. nov. Baranov, OU47576 (holotype); OU47540, OU47572, OU47573, OU47574, OU47575 (paratypes); OU47579, OU47580, OU47581, OU47582 (associated material). Morphotype 1, cf. *Bryophaenocladius*, OU47573, OU47574. *Gymnometriocnemus (G.) extinctus* sp. nov. Baranov, OU47546 (holotype); OU47577, OU47578 (paratypes).

The dataset including the original tiff-images (scaled-down as described above), pvl.nc file and a movie of the specimens are available at zenodo repository on 10.5281/zenodo.14517316.

### New Species Registration

The following information was supplied regarding the registration of a newly described species:

Publication LSID: urn:lsid:zoobank.org:pub:1B39CC4B-AA24-4D9F-819B-F1834E615C5C.

*Bryophaenocladius zealandiae* LSID: urn:lsid:zoobank.org:act:2FE3D4EA-5F0B-4BB4-A2CC-E88557CE825D.

Pterosis extinctus LSID: urn:lsid:zoobank.org:act:DBC38BCD-7C3A-489A-8B47-344576745488.

## References

[ref-1] Albu P, Brundin L (1974). A new subgenus of the genus *Bryophaenocladius* and two new species (Diptera, Chironomidae). Proceedings 5th International Symposium on Chironomidae, Abisko, 7–9 August 1973. Entomologisk Tidskrift Suppl.

[ref-2] Andersen T, Schnell ØA (2000). New species of *Bryophaenocladius* Thienemann, 1934 from Tanzania, with Bare Squama (Diptera: Chironomidae). Aquatic Insects.

[ref-3] Armitage PD (1987). A new species of the genus *Bryophaenocladius* Thienemann, (Diptera: Chironomidae) from Tenerife, Canary Islands. Aquatic Insects.

[ref-4] Armitage PD, Cranston PS, Pinder LCV (1995). The Chironomidae. Biology and Ecology of Non-biting Midges.

[ref-5] Ashe P, O’Connor JP (2012). A world catalogue of Chironomidae (Díptera). Part 2. Orthocladiinae. Two volumes (Sections A, B).

[ref-6] Baranov V, Andersen T, Hagenlund L (2015). A new species of *Bryophaenocladius* Thienemann, 1934 (Diptera, Chironomidae, Orthocladiinae) from Baltic amber. Norwegian Journal of Entomology.

[ref-7] Baranov VO, Haug JT, Kaulfuss U (2024). New records of immature aquatic Diptera from the Foulden Maar Fossil-Lagerstätte, New Zealand, and their biogeographic implications. PeerJ.

[ref-8] Baranov V, Hoffeins C, Hoffeins HW, Haug JT (2019). More than dead males: reconstructing the ontogenetic series of terrestrial non-biting midges from the Eocene amber forest. Bulletin of Geosciences.

[ref-9] Beu AG, Maxwell PA (1990). Cenozoic Mollusca of New Zealand. New Zealand Geological Survey Paleontological Bulletin.

[ref-10] Boothroyd I, Forsyth D (2011). Checklist of New Zealand Chironomidae (Diptera). https://www.chironomidae.net/chklists/New%20Zealand%20Chironomidae%20Checklist%20r10-30-2013.pdf.

[ref-11] Brundin L (1956). Zur Systematik der Orthocladiinae (Diptera, Chironomidae).

[ref-12] Brundin L (1966). Transantarctic relationships and their significance as evidenced by chironomid midges. Kungliga Svenska Vetenskapsakademiens Handligar.

[ref-13] Cookson IC (1947). Plant microfossils from the lignites of Kerguelen Archipelago. British Australian and New Zealand Antarctic Research Expedition 1929–1931, Reports, Series.

[ref-14] Cranston PS (1987). A non-biting midge (Diptera: Chironomidae) of horticultural significance. Bulletin of Entomological Research.

[ref-15] Cranston PS (1996). Identification guide to the Chironomidae of New South Wales. AWR Identification Guide Number 1.

[ref-16] Cranston PS, Hardy NB, Morse GE (2012). A dated molecular phylogeny for the Chironomidae (Diptera). Systematic Entomology.

[ref-17] Cranston PS, Oliver DR, Sæther OA (1989). The adult males of Orthocladiinae (Diptera: Chironomidae) of the Holarctic region—Keys and diagnoses. In: Wiederholm T, ed. Chironomidae of the Holarctic Region. Keys and Diagnoses. Part 3. Adult Males. Entomologica Scandinavica Supplement.

[ref-18] Deevey ES (1955). Paleolimnology of the upper swamp deposit, Pyramid Valley. Records of the Canterbury Museum.

[ref-19] Dieffenbacher-Krall AC, Vandergoes MJ, Woodward CA, Boothroyd IK (2008). Guide to identification and ecology of New Zealand subfossil chironomids found in lake sediment. http://www.climatechange.umaine.edu/Research/facilities/perl/nzguide.html.

[ref-20] Du J, Wang XH, Sæther O (2011). Redescriptions of species of *Bryophaenocladius* Thienemann, 1934 (Diptera: Chironomidae) described by Brundin (1947). Zootaxa.

[ref-21] Edwards FW (1932a). Faune de France: 23. Diptères: Chironomidae, IV. Par M. Goetghebuer. Paris (Lechevalier), 1932. Entomologist.

[ref-22] Edwards FW (1932b). Introduced Diptera in hot-houses at Kew. Entomologist.

[ref-23] Epler JH (2012). A brachypterous *Bryophaenocladius* (Diptera: Chironomidae: Orthocladiinae) with hypopygium inversum from Heggie’s Rock, Georgia, USA. Zootaxa.

[ref-24] Freeman P, Hanström B, Brinck P, Rudebeck G (1955). Diptera (Nematocera): Chironomidae. South African Animal Life.

[ref-25] Freeman P (1959). A study of the New Zealand Chironomidae (Diptera, Nematocera). Bulletin British Museum (Natural History), Entomology.

[ref-26] Freeman P (1961). The Chironomidae (Diptera) of Australia. Australian Journal of Zoology.

[ref-27] GNS Science & Geological Society of New Zealand (2024). New Zealand Fossil Record File [G43/f8500, G45/f0107].

[ref-28] Goetghebuer M (1921). Chironomides de Belgique et spécialement de la zone des Flandres. Mémoires du Musée Royal d’Histoire Naturelle de Belgique.

[ref-29] Goetghebuer M (1932). Diptères Chironomidae IV. (Orthocladiinae, Corynoneurinae, Clunioninae, Diamesinae). Faune de France.

[ref-30] Goetghebuer M (1943). Faunule diptérologique des bois, en Flandre. Biologisch Jaarboek.

[ref-31] Hazra N, Das N (2011). A new species of *Bryophaenocladius* Thienemann, 1934 (Diptera: Chironomidae) from Darjeeling Himalayas, India. International Journal of Dipterological Research.

[ref-32] Hennig W (1960). Die Dipteren-Fauna von Neuseeland als systematisches und tiergeographisches Problem. Beiträge zur Entomologie (= Contributions to Entomology).

[ref-33] Hennig W (1966). Phylogenetic Systematics.

[ref-34] Holmgren AE (1869). Bidrag til Kännedomen om Beeren Eilands och Spetsbergens Insekt-Fauna. Kungliga Svenska Vetenskapsakademiens Handlingar.

[ref-35] Kaczorowska E, Giłka W (2002). The first record of *Bryophaenocladius vernalis* [Goetghebuer, 1921] [Diptera: Chironomidae] in Poland. Polskie Pismo Entomologiczne.

[ref-36] Kaulfuss U, Szawaryn K, Lee DE, Ruta R (2024). The first beetle species described from Oligocene New Zealand amber (Coleoptera: Scirtidae). Palaeoentomology.

[ref-37] Kieffer JJ (1906). Description de nouveaux Diptères Nématocères d’Europe. Annales de la Société Scientifique de Bruxelles.

[ref-38] Kieffer JJ (1911). Nouveaux Tendipédides du groupe Orthocladius [Dipt.]. (2me note). Bulletin de la Société Entomologique de France.

[ref-39] Krosch MN, Baker AM, Mather PB, Cranston PS (2011). Systematics and biogeography of the Gondwanan Orthocladiinae (Diptera: Chironomidae). Molecular Phylogenetics and Evolution.

[ref-40] Krosch M, Cranston PS (2013). Not drowning, (hand) waving? Molecular phylogenetics, biogeography and evolutionary tempo of the ‘Gondwanan’ midge *Stictocladius* Edwards (Diptera: Chironomidae). Molecular Phylogenetics and Evolution.

[ref-41] Krosch MN, Cranston PS, Baker AM, Vink S (2015). Molecular data extend Australian *Cricotopus* midge (Chironomidae) species diversity, and provide a phylogenetic hypothesis for biogeography and freshwater monitoring. Zoological Journal of the Linnean Society.

[ref-42] Krzemiński W, Jarzembowski E (1999). *Aenne triassica* sp. n., the oldest representative of the family Chironomidae (Insecta: Diptera). Polish Journal of Entomology.

[ref-43] Langton PH, Pinder LCV (2007). Keys to the adult male Chironomidae of Britain and Ireland.

[ref-44] Lee D, Lindqvist J, Mildenhall D, Bannister J, Kaulfuss U, Turnbull IM (2009). Paleobotany, palynology and sedimentology of Late Cretaceous—Miocene sequences in Otago and Southland. Field Trip Guides, Geosciences 09 Conference.

[ref-45] Limaye A (2012). Drishti: a volume exploration and presentation tool. Developments in X-ray Tomography VIII. Proceedings of the Society of Photo-Optical Instrumentation Engineers (SPIE).

[ref-46] Lin X, Qi X, Wang X (2012). Two new species of *Bryophaenocladius* Thienemann, 1934 (Diptera, Chironomidae) from China. ZooKeys.

[ref-47] Lindqvist JK, Gard HJL, Lee DE (2016). Geological setting, sedimentology and biota of the estuarine late Oligocene Pomahaka Formation, New Zealand. New Zealand Journal of Geology and Geophysics.

[ref-48] Lytaev P, Hipp A, Lottermoser L, Herzen J, Greving I, Khokhriakov I, Meyer-Loges S, Plewka J, Burmester J, Caselle M, Vogelgesang M, Chilingaryan S, Kopmann A, Balzer M, Schreyer A, Beckmann F (2014). Characterization of the CCD and CMOS cameras for grating-based phase-contrast tomography. Developments in X-Ray Tomography.

[ref-49] Makarchenko EA, Makarchenko MA, Lelei AS (2006). Chironomidae // Key to the insects of Russian Far East. Diptera and Siphonaptera. Part 4.

[ref-50] Marshall S, Kirk-Spriggs HA, Mullerm BS, Paiero MS, Yau T, Jackson MD, Kirk-Spriggs AH, Sinclair BJ (2017). Key to Diptera families—adults. Manual of Afrotropical Diptera. Introductory Chapters and Keys to Diptera Families.

[ref-51] Meigen JW (1830). Systematische Beschreibung der bekannten europäischen zweiflügeligen Insekten.

[ref-52] Moller Pillot HKM (2013). Chironomidae Larvae, Volume 3: biology and ecology of the aquatic Orthocladiinae.

[ref-53] Moosmann J, Ershov A, Weinhardt V, Baumbach T, Prasad MS, LaBonne C, Xiao X, Kashef J, Hoffmann R (2014). Time-lapse X-ray phase-contrast microtomography for in vivo imaging and analysis of morphogenesis. Nature Protocols.

[ref-54] Moubayed J, Langton P (2023). On the genus *Bryophaenocladius* Thienemann, 1934 (Diptera: Chironomidae, Orthocladiinae). II. Description of three new species from continental France. Euroasian Entomological Journal.

[ref-55] Moubayed J, Lods-Crozet B (2022). On the genus *Bryophaenocladius* Thienemann, 1934. I. Taxonomic notes with description of new species (Diptera: Chironomidae, Orthocladiinae). Euroasian Entomological Journal.

[ref-56] Pinder LCV, Armitage PD (1986). The male and female of *Bryophaenocladius muscicola* (Kieffer), based on new material from England (Diptera: Chironomidae). Insect Systematics & Evolution.

[ref-57] Pocknall DT (1982). Palynology of late Oligocene Pomahaka Estuarine Bed sediments, Waikoikoi, Southland, New Zealand. New Zealand Journal of Botany.

[ref-58] Sadowski E-M, Schmidt AR, Seyfullah LJ, Solórzano-Kraemer MM, Neumann C, Perrichot V, Hamann C, Milke R, Nascimbene PC (2021). Conservation, preparation and imaging of diverse ambers and their inclusions. Earth-Science Reviews.

[ref-59] Sæther OA (1973). Four species of *Bryophaenocladius* Thien., with notes on other Orthocladiinae (Diptera: Chironomidae). The Canadian Entomologist.

[ref-60] Sæther OA (1977). Female genitalia in Chironomidae and other Nematocera: morphology, phylogenies, keys. Bulletin of the Fisheries Research Board of Canada.

[ref-61] Sæther OA (1980). Glossary of chironomid morphology terminology (Diptera: Chironomidae). Entomologica Scandinavica Supplement.

[ref-62] Sæther OA (1982). Orthocladiinae (Diptera: Chironomidae) from SE U.S.A., with descriptions of *Plhudsonia*, *Unniella* and *Platysmittia* n. genera and *Atelopodella* n. subgen. Entomologica Scandinavica (= Insect Systematics & Evolution).

[ref-63] Sæther OA (1983). A review of Holarctic *Gymnometriocnemus* Goetghebuer, 1932, with the description of *Raphidocladius* subgen. n. and *Sublettiella* gen. n. (Diptera: Chironomidae). Aquatic Insects.

[ref-64] Sanmartin I, Enghoff H, Ronquist F (2001). Patterns of animal dispersal, vicariance and diversification in the Holarctic. Biological Journal of the Linnean Society.

[ref-65] Schakau B (1991). Stratigraphy of the fossil Chironomidae (Diptera) from Lake Grasmere, South Island, New Zealand, during the last 6000 years. Hydrobiologia.

[ref-66] Schindelin J, Arganda-Carreras I, Frise E, Kaynig V, Longair M, Pietzsch T, Preibisch S, Rueden C, Saalfeld S, Schmid B, Tinevez J-Y, White DJ, Hartenstein V, Eliceiri K, Tomancak P, Cardona A (2012). Fiji: an open-source platform for biological-image analysis. Nature Methods.

[ref-67] Schmidt AR, Kaulfuss U, Bannister JM, Baranov V, Beimforde C, Bleile N, Borkent A, Busch A, Conran JG, Engel MS, Harvey M, Kennedy EM, Kerr PH, Kettunen E, Kiecksee AP, Lengeling F, Lindqvist JK, Maraun M, Mildenhall DC, Perrichot V, Rikkinen J, Sadowski E-M, Seyfullah LJ, Stebner F, Szwedo J, Ulbrich P, Lee DE (2018). Amber inclusions from New Zealand. Gondwana Research.

[ref-68] Seredszus F, Wichard W (2007). Fossil chironomids (Insecta, Diptera) in Baltic amber. Palaeontographica Abteilung A: Paläozoologie, Stratigraphie.

[ref-69] Solórzano-Kraemer MM, Delclòs X, Clapham ME, Arillo A, Peris D, Jäger P, Stebner F, Peñalver E (2018). Arthropods in modern resins reveal if amber accurately recorded forest arthropod communities. Proceedings of the National Academy of Sciences of the United States of America.

[ref-70] Spärck R, Thienemann A (1924). “Metriocnemus” ampullaceus var. austriacus. Verhandlungen der Internationalen Vereinigung für Theoretische und Angewandte Limnologie.

[ref-71] Strenzke K (1957). Terrestrische Chironomiden. XVI. *Bryophaenocladius nitidicollis* (Goetgh.) (Diptera: Tendipedidae, Orthocladiinae). Beiträge zur Entomologie (= Contributions to Entomology).

[ref-72] Stur E, Ekrem T (2015). A review of Norwegian *Gymnometriocnemus* (Diptera, Chironomidae) including the description of two new species and a new name for *Gymnometriocnemus volitans* (Goetghebuer) sensu Brundin. ZooKeys.

[ref-73] Sublette JE, Wirth WW (1980). The Chironomidae and Ceratopogonidae (Diptera) of New Zealand’s subantarctic islands. New Zealand Journal of Zoology.

[ref-74] Trewick SA (2000). Molecular evidence for dispersal rather than vicariance as the origin of flightless insect species on the Chatham Islands, New Zealand. Journal of Biogeography.

[ref-75] van der Wulp FM (1874). Dipterologische aanteekeningen. N°. 4 [Dipterological notes. No. 4.]. Tijdschrift voor Entomologie.

[ref-76] Van Aarle W, Palenstijn WJ, Cant J, Janssens E, Bleichrodt F, Dabravolski A, De Beenhouwer J, Batenburg KJ, Sijbers J (2016). Fast and flexible X-ray tomography using the ASTRA toolbox. Optics Express.

[ref-77] Van Aarle W, Palenstijn WJ, De Beenhouwer J, Altantzis T, Bals S, Batenburg KJ, Sijbers J (2015). The ASTRA toolbox: a platform for advanced algorithm development in electron tomography. Ultramicroscopy.

[ref-78] Veltz I, Azar D, Nel A (2007). New chironomid flies in Early Cretaceous Lebanese amber (Diptera: Chironomidae). African Invertebrates.

[ref-79] Wang X, Andersen T, Sæther OA (2006). Neotropical *Bryophaenocladius* Thienemann, 1934 (Diptera: Chironomidae). Studies on Neotropical Fauna and Environment.

[ref-80] Wang XH, Liu Z, Epler JH (2004). New species of *Bryophaenocladius* Thienemann from the Nearctic Region (Diptera: Chironomidae: Orthocladiinae). Zootaxa.

[ref-81] Wang XH, Sæther OA, Andersen T (2001). Afrotropical *Bryophaenocladius* Thienemann, 1934 (Diptera: Chironomidae). Studia Dipterologica.

[ref-82] Willassen E (1996). A nival *Bryophaenocladius* THIENEMANN, 1934, with reduced wings. Annalen des Naturhistorischen Museums in Wien.

[ref-83] Wood BL (1956). The geology of the Gore subdivision. Gore Sheet District (S170). New Zealand Geological Survey Bulletin.

[ref-84] Woodward CA, Shulmeister J (2007). Chironomid-based reconstructions of summer air temperature from lake deposits in Lyndon Stream, New Zealand spanning the MIS 3/2 transition. Quaternary Science Reviews.

[ref-85] Zelentsov NI, Baranov VA, Perkovsky EE, Shobanov NA (2012). First records on non-biting midges (Diptera: Chironomidae) from the Rovno amber. Russian Entomological Journal.

